# Toxic responses of the liver and kidneys following occupational exposure to anesthetic gases

**DOI:** 10.17179/excli2019-1911

**Published:** 2020-03-25

**Authors:** Masoud Neghab, Fatemeh Amiri, Esmaeel Soleimani, Saeed Yousefinejad, Jafar Hassanzadeh

**Affiliations:** 1 Department of Occupational Health Engineering, Research Center for Health Sciences, Institute of Health, School of Health, Shiraz University of Medical Sciences, Shiraz, Iran; 2 Department of Occupational Health Engineering, Social Determinants in Health Promotion Research Center, Hormozgan Health Institute, Hormozgan University of Medical Sciences, Bandar Abbas, Iran; 3 Department of Occupational Health Engineering, School of Health, Shiraz University of Medical Sciences, Shiraz, Iran; 4 Department of Clinical Epidemiology, School of Health, Shiraz University of MedicalSciences, Shiraz, Iran

**Keywords:** inhalation anesthetics, occupational exposure, biological monitoring, hepatotoxicity, nephrotoxicity

## Abstract

This study was undertaken to determine whether exposure of operating room personnel to inhalation anesthetics, including nitrous oxide, isoflurane, and sevoflurane was associated with any hepatotoxic or nephrotoxic changes. Fifty-two operating room personnel and 52 non-exposed subjects were studied. A questionnaire pertaining to demographic characteristics and medical history of participants was completed. Fasting blood samples were taken from all subjects to measure the functional parameters of kidneys and liver. Biological monitoring was also performed to detect the urinary concentration of IAs. Urinary concentrations of nitrous oxide, isoflurane, and sevoflurane were found to be 175.8 ± 77.52, 4.95 ± 3.43, and 15.0 3± 16.06 ppm, respectively. The mean levels of alanine aminotransferase, aspartate aminotransferase, alkaline phosphatase, gamma-glutamyltransferase, Alpha-glutathione-S-transferase, as well as the serum levels of kidney injury molecule-1, creatinine and calcium were significantly higher in the exposed group. Statistically significant associations were observed between exposure to inhalation anesthetics and the mean levels of aspartate aminotransferase, alanine aminotransferase, and gamma-glutamyltransferase, serum creatinine, kidney injury molecule-1, and calcium. Under the exposure scenario described in the present study, occupational exposure to inhalation anesthetics was associated with subtle, subclinical, pre-pathologic changes in the parameters of liver and kidneys. Additionally, Alpha-glutathione-S-transferase and kidney injury molecule-1 were found to be sensitive markers for early detection of subclinical changes in the parameters of kidney and liver function in subjects who are exposed to inhalation anesthetics.

## Abbreviations

BMI body mass index

IA inhalation anesthetics

Alpha-GST alpha-glutathione-S-transferase

ALP alkaline phosphatase

ALT alanine aminotransferase

ASP aspartate aminotransferase

AST aspartate aminotransferase

BUN blood urea nitrogen

GGT gamma-glutamyltransferase

KFT kidney function tests

KIM-1 kidney injury molecule-1

LFT liver function tests

ORP operating room personnnel

WAG waste anesthetic gases

## Introduction

Anesthetic gases or inhalation anesthetics (IAs) are widely used for the induction and maintenance of anesthesia during surgery (Steffey et al., 2015[45]). Nitrous oxide, isoflurane, sevoflurane, and desflurane are common IAs used for anesthesia (CCOHS, 2017[10]). In Iran, nitrous oxide, isoflurane and sevoflurane are the most common used IAs in general anesthesia (Amiri et al., 2018[5]). Besides their importance in medicine, IAs are considered as an important chemical hazard for operating room personnel (ORP) (Accorsi et al., 2001[1]). Leakages of IAs from the patient's anesthetic breathing circuit into the air of operating rooms during delivery of anesthesia are known as waste anesthetic gases (WAGs) (NIOSH, 2007[33]). Leakages may occur in several situations such as a) poor connection of connectors, tubing, and valves, b) escaping IAs during hookup and disconnection of the system, and c) leaking IAs slowly over the lip of the patient's mask or from endotracheal coupling during pediatric anesthesia. Such situations pose a risk of occupational exposure of ORP to WAGs (Braz et al., 2017[7]; NIOSH, 2007[33]).

It is estimated that more than 200,000 health care workers in the United States are potentially exposed to WAGs in their workplace and are at the risk of developing adverse health effects due to long-term exposure to these agents (OSHA, 2000[36]). Acute (short-term) exposure to high concentrations of WAGs is associated with adverse health outcomes such as headache, fatigue, drowsiness, and difficulties with judgment and coordination (NIOSH, 2007[33]). On the other hand, chronic (long-term) exposure to WAGs may result in reproductive and developmental effects, such as spontaneous abortion, birth defects, infertility (Nagella et al., 2015[30]; Mohammed, 2016[29]); genotoxicity (Yilmaz and Calbayram, 2016[52]; Shouroki et al., 2019[41]), as well as hematotoxicity (Amiri et al., 2018[5]), hepatotoxicity (Safari et al., 2014[38]; Nicoll et al., 2012[31]; Iaizzo et al., 1990[19]) and nephrotoxicity (Jafari et al., 2018[22]). However, hepatotoxicity and nephrotoxicity potentials of chronic exposure to WAGs is subject of debate and controversy.

While in some studies exposure to WAGs has been shown to be associated with hepatotoxic and nephrotoxic outcomes (Safari et al., 2014[38]; Caciari et al., 2013[8]; Nicoll et al., 2012[31]; Iaizzo et al., 1990[19]; ASA, 1974[6]), other studies have failed to demonstrate such an association (Ong Sio et al., 2017[35]; Nishiyama, 2013[34]; Sun et al., 1997[48]; Soma et al., 1995[44]; Franco et al., 1993[14]; Guirguis et al., 1990[17]). For instance, an increased risk of liver and kidney diseases has been reported among subjects exposed to anesthetic gases as compared with a non-exposed group (ASA, 1974[6]). Similarly, Caciari et al. reported a relationship between chronic exposure to low levels of anesthetic gases and changes in the parameters of kidney and liver function (Caciari et al., 2013[8]). In contrast, in a study of health effects associated with exposure to anesthetic gases in Ontario hospital personnel, Guirguis et al. did not find any association between exposure to IAs and liver or kidney diseases (Guirguis et al., 1990[17]). 

Although the exact reason(s) for these discrepancies are not known, they could be explained, at least in part, by differences in the type and concentrations of anesthetic gases, length of exposure, co-exposure to other chemicals, type of studies, sample size, statistical analyses, and shortcomings, such as lack of control for potential confounders and selection of inappropriate control groups.

The present study was undertaken to examine, more thoroughly, whether exposure to WAGs, including nitrous oxide, isoflurane, and sevoflurane, by a group of ORP, under their usual working condition, is associated with any significant changes in the conventional and/or more sensitive parameters of kidney and liver function.

## Materials and Methods

### Subjects and study design

In this historical cohort study, hepatotoxic and nephrotoxic effects of chronic exposure to WAGs, including nitrous oxide, isoflurane, and sevoflurane were assessed in a group of ORP at a large public hospital in Shiraz, south of Iran. The exposed group composed of 52 ORP (anesthesiologists, surgeons, surgical technicians, and nurses) with a history of at least one-year exposure to the WAGs. Similarly, 52 unexposed employees from administrative staff were randomly selected and served as the referent group.

A questionnaire pertaining to demographic characteristics and medical history, including age, sex, level of education, smoking habits, alcohol consumption, duration of employment (exposure), mental and physical diseases, history of exposure to hepatotoxic and/or nephrotoxic chemicals, and previous or current history of kidney and liver diseases was completed for all subjects. Individuals with a history of renal and liver disease, history of taking hepatotoxic and nephrotoxic drugs, and those with previous or current exposure (occupational or non-occupational) to other known hepatotoxic and nephrotoxic agents were excluded from the study.

Subjects signed an informed consent before participating in the study. The protocol of the study was approved by the ethics committee of Shiraz University of Medical Sciences (IR.SUMS.RE C.1396.S388) and the study was conducted in accordance with the Helsinki declaration of 1964 as revised in 2013.

### Liver and kidney function tests

Fasting blood samples (5 ml) were taken from the antecubital vein of the studied subjects at their workplace. Clot blood samples were centrifuged at 3500 rpm for 5 minutes to obtain the sera and stored at -80 °C until analysis. Serum activities of alanine aminotransferase (ALT), aspartate aminotransferase (AST), alkaline phosphatase (ALP), gamma-glutamyltransferase (GGT), albumin, total protein, total bilirubin, direct bilirubin, blood urea nitrogen (BUN), creatinine, calcium, and phosphorus were determined by colorimetric methods using commercially available diagnostic kits (Pars Azmoon Co., Karaj, Iran) by an auto analyzer (model BT 1500, Biotecnica Instrument Co, Rome, Italy). The Alpha-glutathione S-transferase (alpha-GST) activity was determined spectrophotometrically according to the method published by Mannervik and Guthenberg (1981). All substrates for the activity measurements and kinetic studies were purchased from Sigma-Aldrich. Serum levels of kidney injury molecule-1 (KIM-1) were measured using the ELISA kit (ZellBio GmbH, Germany), according to the manufacturer's instructions. Serum Potassium level was measured by flame photometry method.

### Biological monitoring

Concentrations of nitrous oxide, isoflurane and sevoflurane were measured in 30 urinary samples taken from anesthesiologists, surgeons, surgical technicians, and nurses. Urine samples were collected at the end of the morning operating shift (after at least three hours of exposure). About 10 ml of a urine sample was transferred with a disposable Luer lock syringe to a 20 ml screw cap headspace vial pre-sealed with a PTFE/ rubber septum. Samples were kept on ice packs in a cold box and immediately transported to the laboratory where they were analyzed. Analysis of the urine samples was performed in accordance with a previously published method (Accorsi et al., 2001[1]).

### Instrumentation 

Urine samples were analyzed using an Agilent 5977B gas chromatography-mass spectrometry (GC/MS) coupled with an Agilent 7697A headspace auto-sampler. The separation was performed in an Hp-5ms capillary column (30 m × 0.25 mm × 0.25 µm) (Agilent, Palo Alto, Calif. USA). The injection block temperature was set at 250 °C. Initial oven temperature was 40 °C for 4 min, followed by an increase of 40 °C /min to 140 °C. The injection volume was 1 µl (split 1:2). Helium was used as the carrier gas at a flow rate of 1.2 mL/min. MS interface temperature was set at 250 °C. Headspace loop, vials equilibrium, and transfer line temperature were set at 60, 42, and 70 °C, respectively. The running time was 10 min. A solvent delay time was 0.5 min. Quantification in MS was performed in selected ion monitoring (SIM) mode at a mass-to-charge ratio (m/z) of 30 for nitrous oxide*, *51 for isoflurane*, *and 131 for sevoflurane. Limits of detection for nitrous oxide, isoflurane, and sevoflurane were 10.31, 0.63, and 0.17 μg/l, respectively.

### Statistical analyses

All statistical analyses were performed using version 20.0 of statistical package for social sciences (SPSS) software. Descriptive results are presented as arithmetic mean ± SD. Independent sample t-test and Chi-square were used for comparing the means of quantitative and qualitative variables, respectively. Multiple linear regression analysis was used to adjust the effects of confounding variables, such as age, sex, and body mass index (BMI) on the relationships between exposure to WAGs and the parameters of kidney function tests (KFT) and liver function tests (LFT).

## Results

Demographic characteristics of the studied subjects are shown in Table 1(Tab. 1). The mean ages of the exposed and non-exposed subjects were 34.19 ± 5.82 and 33.73 ± 6.84 years, respectively. Only one subject (2 %) in the exposed group and two (4 %) subjects in the non-exposed group were smokers. No significant differences were observed between the groups as far as their demographic characteristics were concerned. Urinary concentrations of nitrous oxide, isoflurane, and sevoflurane in the ORP were 175.8 ± 77.52 (range: 7.98-319.91), 4.95 ± 3.43 (range: 0.78-14.9), and 15.03 ± 16.06 ppm (range: 0.76-46.40 ppm), respectively. 

The results of the KFT and LFT of the studied subjects are shown in Table 2(Tab. 2). The mean values of AST, ALT, GGT, alpha-GST, creatinine, KIM-1, and calcium were significantly higher in the exposed group than in the non-exposed subjects. In contrast, no significant differences were observed between the groups for serum albumin, total proteins, ALP, direct bilirubin, total bilirubin, BUN, potassium and phosphorus. 

The associations between exposure to the WAGs and the parameters of liver and kidney function tests are shown in Tables 3(Tab. 3) and 4(Tab. 4). As seen, after adjusting for the effects of confounding variables of age, sex and body mass index (BMI), statistically significant associations were observed between exposure to WAGs and the mean levels of AST, ALT, and GGT in that exposure to the WAGs resulted in 4.79, 7.9, 8.58, and 0.55 units increments in the levels of AST, ALT, GGT and alpha-GST, respectively (Table 3(Tab. 3)). The parameters of kidney function were less affected than those of liver function. Exposure to WAGs was associated with slight but significant increments in the levels of serum creatinine, KIM-1, and calcium (0.07, 0.22, and 0.58 units, respectively) (Table 4(Tab. 4)).

## Discussion

In this historical cohort study, possible hepatotoxic and nephrotoxic effects of chronic exposure to nitrous oxide, isoflurane and sevoflurane were studied in a group of ORP. No significant differences were noted between the exposed and referent subjects as far as their demographic characteristics were concerned. Additionally, none of the studied subjects had a history of exposure to hepatotoxic and/or nephrotoxic substances.

The American Conference of Governmental Industrial Hygienists (ACGIH) has proposed the TLV values of 50 and 5 ppm for nitrous oxide and isoflurane respectively (ACGIH, 2019[3]). Also, NIOSH has recommended REL values of 2 ppm for all the halogenated agents and 25 ppm for nitrous oxide (NIOSH, 1994[32]). Although BEI levels for these anesthetic gases have not been established by ACGIH, Imbriani et al. (1995[21]) have proposed that urinary level of nitrous oxide equal to 25 μg/l corresponds with 50 ppm (TLV) of this gas in the air. Similarly, urinary concentration of 5.6 μg/l of isoflurane corresponds with 2 ppm (TLV) of this gas in the air. Similarly, Accorsi et al. have reported biological equivalent limits of 35.5 and 22.3 μg/l for nitrous oxide corresponding with the ACGIH and NIOSH exposure limits, respectively, and 3.6 μg/l for sevoflurane based on the NIOSH exposure limit of 2 ppm (Accorsi et al., 2003[2]).

In this study, urinary concentrations of nitrous oxide, isoflurane, and sevoflurane in the ORP were found to be 175.8 ± 77.53, 4.95 ± 3.43, and 15.03 ± 16.06 ppm, respectively. The IAs were not detectable in the urine of the non-exposed group. These concentrations, quantitatively, correspond with the findings of Kargar et al. for anesthetic gases in the air. They have shown that environmental exposures of ORP to these agents were higher than national recommended exposure limits (Shouroki et al., 2019[41]). 

The mean concentration of nitrous oxide in urine was about 7 and 5 fold higher than the limits set by Imbriani et al. (1995[21]) and Accorsi et al. (2003[2]), respectively. The mean value determined for sevoflurane in this study was about 4 fold higher than the limit set by Accorsi et al. (2003[2]). Biological equivalent limit for isoflurane set by Imbriani et al. (2003[21]) was slightly higher than the value determined in our study. In line with these observations, Accorsi et al. (2001[1]) analyzed post-shift urine of operating room staff by gas chromatography-mass spectrometry coupled with headspace sampling. Levels of anesthetic gases in the urine of subjects ranged from 0.4 to1415.9 μg/l for nitrous oxide, 0.0 to 36 μg/l for isoflurane, and 0.0 to 46 μg/l for sevoflurane. Similarly, Al-Ghanem et al. measured the concentration of anesthetic gases in the urine of forty operating room personnel. The mean urinary concentrations of nitrous oxide, isoflurane, and sevoflurane were 1234, 3.75, and 4.3 μg/l, respectively (Al-Ghanem et al., 2008[4]). Other studies reported urinary levels of isoflurane and sevoflurane following occupational exposure to these compound as 2.42 ± 2.86 (range: 0.31-13.38) and 0.006 ± 3.83 (range: non-detected -2.41), respectively (Jafari et al., 2018[22]).

The levels of exposure to WAGs depend on several parameters, including absence or presence of proper ventilation and scavenging systems in operating rooms, type of surgery, the extent of leaks from anesthesia face masks during the administration of the anesthetic gases to the patients, leakage from cylinders, whether there is a regular check for detecting gas leakage from anesthetic machines and length of daily shifts. Also, inappropriate work practices such as starting anesthetic gas flow before applying a mask on the patient's face or closing the anesthetic gas flow after removing a face mask and poorly fitted face masks play a major role in this scenario.

The finding of the present study revealed that the mean values of all parameters of liver function test for both groups were within the normal range. However, serum AST, ALT, and GGT were significantly higher in the exposed group (Table 2(Tab. 2)) than in the referent individuals. Measurements of serum activities of liver enzymes are the golden standard for anesthetic-related hepatic toxicity. However, conventional serum enzyme tests suffer from low specificity and sensitivity. These markers are also present in other tissues, such as heart, kidney and muscles and consequently may be influenced by non-specific liver damage, including acute viral illnesses, cardiac and skeletal muscle injury. More importantly, these markers only identify later phases or progression of liver injury (Limdi and Hyde, 2003[26]; Koo et al., 2000[25]). Therefore, more sensitive markers, such as alpha-GST activity was also studied among the ORP. Advantages of using alpha-GST as an optimal biomarker of the liver function are a smaller molecular weight and shorter half-life (about 90 min). Also, the serum alpha-GST concentration is rapidly raised following hepatocellular damage (Koo et al., 2000[25]). Unlike aminotransferases, alpha-GST is distributed mainly in the centrilobular region and is secreted into the blood in the case of liver injuries (Mikstacki et al., 2016[28]; Haschek et al., 2013[18]). Yousif et al. demonstrated that the measurement of alpha-GST activity as an indicator of hepatocellular integrity is a more sensitive biomarker than conventional liver enzymes for monitoring of hepatic damage after anesthesia with sevoflurane (Yousif et al., 2009[53]).

The mean serum alpha-GST activity was significantly higher in the exposed group than in the referent individuals. Interestingly, after adjusting for important confounders, statistically significant positive associations were found between exposures to the WAGs and serum activities of ALT, AST, GGT and alpha-GST (Table 3(Tab. 3)). These increases may occur after exposure to low concentrations of many xenobiotic substances. While some researchers consider these to be adaptive reactions, others interpret them as signs of early impairments (Stellman, 1998[46]).

Halogenated anesthetic agents have been reported to cause liver damage. Reported injury has ranged from mild injury to fulminant hepatic failure. Mild injury is characterized with a slight increase in serum activity of aminotransferases while fulminant hepatic failure is associated with the significant increase in the activity of liver enzymes and bilirubin level which lead to severe liver necrosis (Weitz et al., 1997[51]). 

Our findings are in line with the proposition that long-term exposure to IAs (nitrous oxide, isoflurane, and sevoflurane) is associated with subtle, sub-clinical, pre-pathologic changes in both conventional and more sensitive parameters of liver function. Additionally, it is in agreement with the findings of some experimental and epidemiological studies. For example, Casale et al. (2014[9]) reported in a study of the effects of chronic occupational exposure to low concentrations of anesthetic gases (halothane, enflurane, and isoflurane mixed with nitrous oxide and oxygen) significant changes in serum activities of ALT, AST, GGT and total bilirubin of exposed workers. Ghanbari et al. (2017[15]) also studied 400 people of the nursing team and operating room personnel and found that parameters of liver function including ALT and AST were significantly higher in operating room personnel than in nursing personnel. Similarly, Jafari et al. found significant increases in the serum activity of AST and ALT in 42 ORP exposed to sevoflurane and isoflurane in comparison with a group of 30 healthy hospital personnel (Jafari et al., 2018[22]). In contrast, some investigators have failed to demonstrate that occupational or non-occupational exposure to anesthetic gases were associated with changes in the parameters of liver function. For example, in a study conducted on the employees working at an operating room, no significant difference was noted between the exposed and unexposed participants in terms of liver function test (Franco et al., 1993[14]). Similar results were reported by other authors in operating theatre members (Saurel-Cubizolles et al., 1992[40]). 

These controversial findings suggest that other factors, including duration and severity of exposure, the type of anesthetic gases, age, gender, co-exposure to other chemicals may affect the hepatotoxicity of these agents. Overall, the side effects of anesthetic agents, including nitrous oxide, isoflurane and sevoflurane are dependent on the magnitude and frequency of exposure (Smith, 1998[43]; Jafari et al., 2018[22]). Also, it is known that chronic exposure is mainly a risk factor for hepatotoxicity of halogenated anesthetic gases (Singhal et al., 2010[42]).

Several mechanisms are responsible for hepatic injury following exposure to anesthetic gases. Fassoulaki et al. (1984[13]) reported that exposure to all anesthetic agents is expected to be associated with hepatotoxicity as a result of the decrease in liver blood flow according to the hypoxic rat model. Halogenated agents may also cause liver damage through disruption of cellular calcium homeostasis mechanisms (Turillazzi et al., 2007[50]). As far as results of novel and common parameters of kidney function test, including KIM-1, BUN, creatinine, and serum level of calcium, potassium, and phosphorus were concerned, it is of interest to note that the mean values of kidney tests in both groups were within the normal range. However, despite this observation, one could not simply ignore the fact that the mean values of KIM-1, creatinine, and calcium for the exposed group were significantly higher than those of unexposed subjects. 

The majority of previous studies have assessed the relationship between WAGs and kidney injuries in anesthetized patients and limited studies have investigated such a relationship in subjects occupationally exposed to these chemicals. For instance, Cohen et al. reported a high frequency of kidney disease as a result of chronic occupational exposure to anesthetic gases among operating room staff, especially female (Cohen, 1978[11]). Caciari et al. (2013[8]) reported in their study of 154 operating room staff and 98 control individuals, that occupational exposure to low concentration of anesthetic gases could influence renal parameters in exposed health personnel. In contrast, Trevisan et al. (2003[49]) investigated the effect of nitrous oxide and sevoflurane on renal function of exposed personnel. They showed that renal function biomarkers were not affected by these agents. Similarly, Groudine et al. (1999[16]) reported that exposure to low-flow of isoflurane and sevoflurane are not associated with significant renal effects. Likewise, Saricaoglu et al. (2006[39]) did not find any difference in creatinine levels after sevoflurane and isoflurane anesthesia after elective coronary artery surgery.

Serum creatinine and BUN are two useful indicators for evaluating kidney function. However, their uses as sensitive and specific biomarkers for early detection of kidney injuries have numerous limitations. First, many non-renal factors such as age, sex, race, body weight, diet, and drug consumption can influence serum creatinine level. Second, the production rate of urea is not stable and increases with the consumption of protein-rich diets or under situations such as bleeding, muscle trauma, and/or steroid administration (Klaassen and Amdur, 2013[24]).

Therefore, it may be argued that the elevated creatinine levels observed in this study may not necessarily be attributed to the exposure to the anesthetic gases. While true, the following lines of circumstantial evidence indicate that the observed effects are very likely to be related to exposure to the anesthetic gases. First, KIM-1, as a sensitive and specific biomarker for early detection of renal injury, is not virtually detectable in subjects with healthy kidneys and within hours after kidney injury, its level markedly increased in both animals and humans (Khreba et al., 2019[23]). Therefore, its detection may be evidence of early change of renal function and it can be served as a diagnostic discriminator (Ichimura et al., 1998[20]). Second, regular excretion of calcium and phosphate is primarily done by the kidneys. Renal dysfunction may be associated with impairment of bone turnover and alteration of calcium levels. Therefore, the significantly higher serum calcium level in the studied OPR may be due to potential nephrotoxicity of the WAGs (Dhondup and Qian, 2017[12]).

Finally, as shown in Table 3(Tab. 3), after adjusting for potential confounders significant associations were noted between exposure and calcium, KIM 1 and creatinine. Inherent limitations of cross-sectional studies such as the present one do not allow a cause and effect relationship to be established. However, the following lines of circumstantial evidence support the hypothesis that long-term exposure to these chemicals may be attributed to kidney and/or liver injuries:

1. Subjects were free from any preexisting medical conditions, particularly, liver and renal diseases and did not take any hepatotoxic and/or nephrotoxic drugs. Also, they had no history of exposure to hepatotoxic and nephrotoxic agents.
2. The major organs affected by anesthetic agents (isoflurane and sevoflurane) and their metabolites are the liver and kidneys because they are metabolized through the metabolic pathway involving cytochrome P-450 2E1 in the liver and kidneys (Prout et al., 2014[37]; Stoelting and Miller, 2006[47]).
3. After adjusting for potential confounders, statistically significant associations were noted between long-term exposure to the WAGs and the serum levels of creatinine, KIM-1, and calcium as well as the serum levels of ALT and AST, GGT, and alpha-GST.

Our findings indicate that under the exposure scenario described in this study, occupational exposure to a mixture of WAGs may cause subtle, subclinical, pre-pathologic changes in parameters of liver and kidney function tests. Although the observed disruptions were not clinically significant, the development of pathological changes may arise given the fact that the studied ORP have to work for another 20 years and may be repeatedly exposed to the WAGs. Whether these effects may progress to pathological consequence and the ramifications of these effects deserve further investigations. Alpha-GST and KIM-1 can be considered as sensitive and specific biomarkers of liver and kidney injuries, respectively, which are recommended to assess in the periodic examinations of WAGs-exposed subjects for early detection of liver and kidney disorders.

## Acknowledgement

This work was financially supported by the Shiraz University of Medical Sciences, Vice-Chancellor for Research and Technology (Grant number: 96-01-04-14653) and Iran National Science Foundation (Grant number: 96005391).

## Conflict of interest

The authors declared that they have no conflict of interest.

## Figures and Tables

**Table 1:**
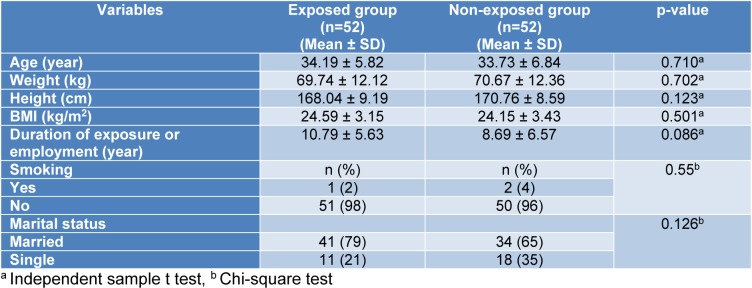
Table1: Demographic characteristics of the studied participants

**Table 2:**
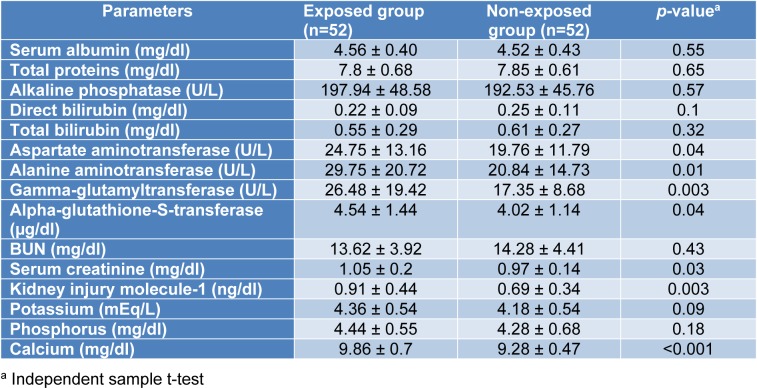
Comparison of kidney and liver function tests between the exposed and non-exposed groups (Mean ± SD)

**Table 3:**
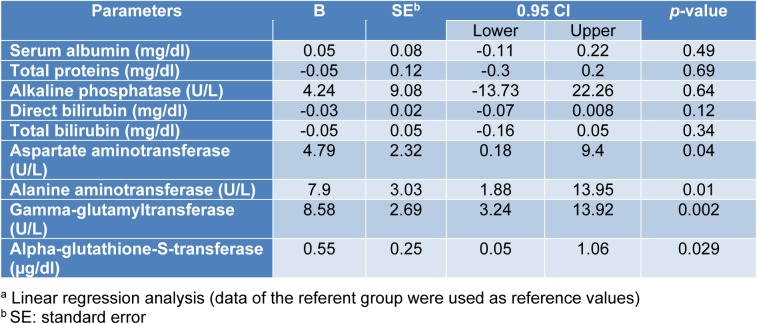
Table3: Association between urinary levels of the WAGs and the parameters of liver function tests^a^

**Table 4:**
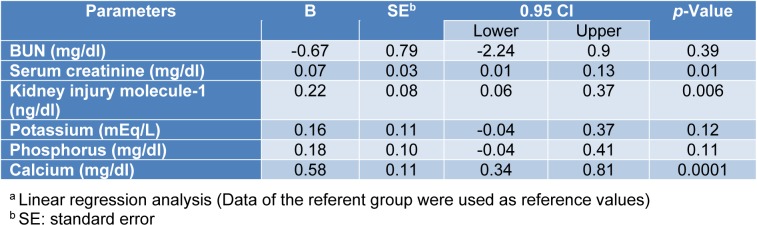
Association between urinary levels of the WAGs and the parameters of kidney function tests^a^
