# Erratum to: Toxic responses of the liver and kidneys following occupational exposure to anesthetic gases

**DOI:** 10.17179/excli2021-3517

**Published:** 2021-02-15

**Authors:** Masoud Neghab, Fatemeh Amiri, Esmaeel Soleimani, Saeed Yousefinejad, Jafar Hassanzadeh

**Affiliations:** 1 Department of Occupational Health Engineering, Research Center for Health Sciences, Institute of Health, School of Health, Shiraz University of Medical Sciences, Shiraz, Iran; 2 Department of Occupational Health Engineering, Social Determinants in Health Promotion Research Center, Hormozgan Health Institute, Hormozgan University of Medical Sciences, Bandar Abbas, Iran; 3 Department of Occupational Health Engineering, School of Health, Shiraz University of Medical Sciences, Shiraz, Iran; 4 Department of Clinical Epidemiology, School of Health, Shiraz University of MedicalSciences, Shiraz, Iran

## ⁯

Erratum to EXCLI Journal 2020;19:418-429 DOI 10.17179/excli2019-1911

418

The authors would like to correct

on page 418 (Abstract):

The unit ppm needs to be changed to ppb. The sentence should read:

“Urinary concentrations of nitrous oxide, isoflurane, and sevoflurane were found to be 175.8 ± 77.52, 4.95 ± 3.43, and 15.0 3± 16.06 **ppb**, respectively”.

On page 421 (Results, first paragraph):

The unit ppm needs to be changed to ppb. The sentence should read:

“Urinary concentrations of nitrous oxide, isoflurane, and sevoflurane in the ORP were 175.8 ± 77.52 (range: 7.98-319.91), 4.95 ± 3.43 (range: 0.78-14.9), and 15.03 ± 16.06 **ppb** (range: 0.76-46.40 **ppb**), respectively.”

On page 423 (Discussion, third paragraph)

The unit ppm needs to be changed to ppb. The sentence should read:

“In this study, urinary concentrations of nitrous oxide, isoflurane, and sevoflurane in the ORP were found to be 175.8 ± 77.52, 4.95 ± 3.43, and 15.03 ± 16.06 **ppb**, respectively”.

